# The Association between Ambient Air Pollution and Allergic Rhinitis: Further Epidemiological Evidence from Changchun, Northeastern China

**DOI:** 10.3390/ijerph14030226

**Published:** 2017-02-23

**Authors:** Bo Teng, Xuelei Zhang, Chunhui Yi, Yan Zhang, Shufeng Ye, Yafang Wang, Daniel Q. Tong, Binfeng Lu

**Affiliations:** 1 Department of Otolaryngology Head and Neck Surgery, The Second Hospital, Jilin University, Changchun, 130041, China; yeshufeng16@163.com (S.Y.); wangyafangwang@163.com (Y.W.); binfeng@pitt.edu (B.L.); 2 Key Laboratory of Wetland Ecology and Environment, Northeast Institute of Geography and Agroecology, Chinese Academy of Sciences, Changchun 130102, China; 3 Center for Spatial Information Science and Systems, George Mason University, Fairfax, VA 22030, USA; tongquansong@neigae.ac.cn; 4 Department of Pathology, Mount Sinai West, New York City, NY 10019, USA; yich1@hotmail.com; 5 Department of Otolaryngology Head and Neck Surgery, The First Hospital, Jilin University, Changchun 130021, China; zhangyan993300@163.com; 6 U.S. NOAA Air Resources Laboratory, College Park, MD 20740, USA; 7 Department of Immunology, University of Pittsburgh School of Medicine, Pittsburgh, PA 15261, USA

**Keywords:** air pollution, allergic rhinitis, significant association, seasonal effect, lag effects, Changchun

## Abstract

With the continuous rapid urbanization process over the last three decades, outdoors air pollution has become a progressively more serious public health hazard in China. To investigate the possible associations, lag effects and seasonal differences of urban air quality on respiratory health (allergic rhinitis) in Changchun, a city in Northeastern China, we carried out a time-series analysis of the incidents of allergic rhinitis (AR) from 2013 to 2015. Environmental monitoring showed that PM_2.5_ and PM_10_ were the major air pollutants in Changchun, followed by SO_2_, NO_2_ and O_3_. The results also demonstrated that the daily concentrations of air pollutants had obvious seasonal differences. PM_10_ had higher daily mean concentrations in spring (May, dust storms), autumn (October, straw burning) and winter (November to April, coal burning). The mean daily number of outpatient AR visits in the warm season was higher than in the cold season. The prevalence of allergic rhinitis was significantly associated with PM_2.5_, PM_10_, SO_2_ and NO_2_, and the increased mobility was 10.2% (95% CI, 5.5%–15.1%), 4.9% (95% CI, 0.8%–9.2%), 8.5% (95% CI, −1.8%–19.8%) and 11.1% (95% CI, 5.8%–16.5%) for exposure to each 1-Standard Deviation (1-SD) increase of pollutant, respectively. Weakly or no significant associations were observed for CO and O_3_. As for lag effects, the highest Relative Risks (RRs) of AR from SO_2_, NO_2_, PM_10_ and PM_2.5_ were on the same day, and the highest RR from CO was on day 4 (L4). The results also indicated that the concentration of air pollutants might contribute to the development of AR. To summarize, this study provides further evidence of the significant association between ambient particulate pollutants (PM_2.5_ and PM_10_, which are usually present in high concentrations) and the prevalence of respiratory effects (allergic rhinitis) in the city of Changchun, located in Northeastern China. Environmental control and public health strategies should be enforced to address this increasingly challenging problem.

## 1. Introduction

Along with the rapid economic growth and urbanization, the severe and deteriorating regional haze has smothered the eastern region of China. The health effects caused by outdoor air pollution have become a sensitive topic for the public, media and even the government of China and adjacent countries. The need for a better comprehension to the role of ambient air pollution on human health and implementing suitable protective policies has fueled related studies in the past decade.

A number of adverse health effects, including non-accidental death, respiratory diseases (such as, rhinitis, asthma, tracheitis, pneumonia), cardiovascular diseases (such as stroke, arrhythmia, ischemic heart disease, cerebrovascular disease), cardiopulmonary diseases (chronic obstructive pulmonary disease, COPD) and, more rarely, conjunctivitis, dermatological disorders, skin allergy and exacerbated cough are associated with ambient air pollution [1]. 

**Figure 1:**
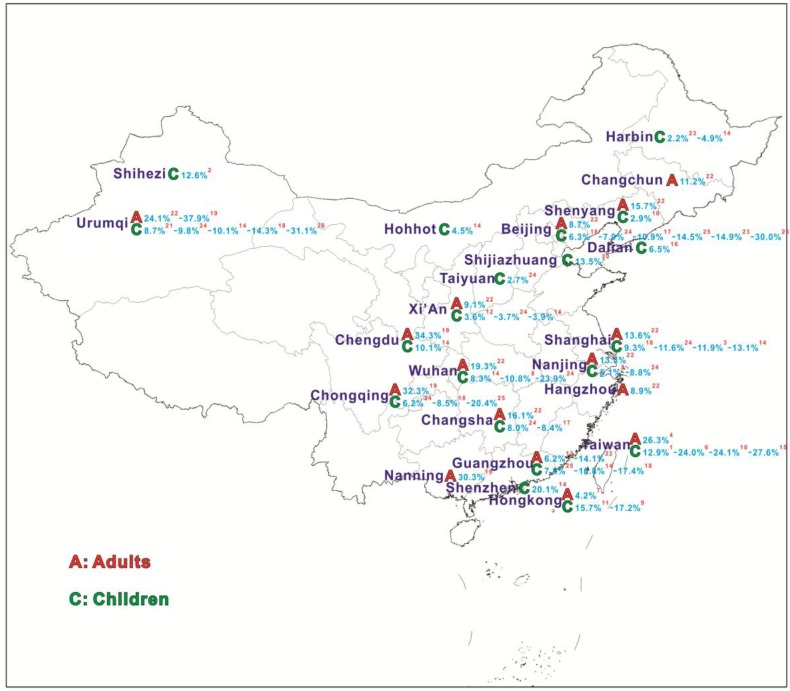
Comprehensive published prevalences of AR in adults and children in different Chinese cities.

Most studies regarding respiratory diseases have addressed asthma and COPD, and only limited studies have focused on allergic rhinitis (AR) in China. As a typical respiratory illness, AR affects 20%–40% of the population worldwide, although the prevalence varies with age and region [2,3]. Although it is usually a minor respiratory disease, AR frequently presented with symptoms that affect work performance and quality of daily life, and consumes health recourses [4]. According to the Allergies in Asia-Pacific Survey, one of the largest studies of AR on adults and children in Asia, the prevalence of AR was 8.7% in Asia. The prevalence of self-reported AR in adults is much lower in China than in many Western and developed/developing countries (such as Japan and Korea). The age- and gender-adjusted incidence of AR was approximately 14% in China, ranging from 8.7% (Beijing) to 24.1% (Urumqi) in Figure 1. The prevalence of AR for adults was 11.2% and 15.7% in Changchun and Shenyang of northeastern China, respectively [5,6]. According to a cross-sectional questionnaire survey during 2010–2012, the prevalence of rhinitis in the 10 cities varied from 2.2% to 23.9% (mean 8.5%) [7].

Globally, studies have shown associations between vehicle and industrial emissions and increased risk of AR [8,9]. Individual pollutants responsible for the increased risk of allergic disease were nitrogen oxides (NO_x_), sulphur dioxide (SO_2_), ozone (O_3_), particulates with an aerodynamic diameter of 10 μm or less (PM_10_), and particulates with an aerodynamic diameter of 2.5 μm or less (PM_2.5_) [10,11]. Contradictory findings also have been found for the SO_2_, NO_2_, O_3_ and PM_10_ levels in some other studies for children and the elderly [7,12]. Both cross-sectional and cohort studies have shown obvious associations between traffic NO_2_ pollution and AR in children [8,13]. Ozone was also associated with AR in children who reside in industrial areas [9]. Air pollutants, such as PM_10_, PM_2.5_, O_3_, CO, NO, NO_2,_ and SO_2_ had positive correlation with AR incidents, when compared with meteorological factors in a heavy industry area of northern Taiwan [14]. There was no association between mean level of pollutants (SO_2_, NO_2_, O_3_) and symptoms of Ear, Nose, and Throat (acute rhinitis, 12-month rhinitis, fever, rhinoconjunctivitis, and hay fever) in children. Furthermore, several studies have also demonstrated that air pollution can promote and exaggerate response to allergens in the nasal cavity by increasing the allergenicity and bioavailability of airborne pollen allergens [15].

However, for the ambient pollutants, only six studies reports on the influence of outdoor air pollutant and the prevalence of AR in Asia [16]. A nationwide cross-sectional study covering Taiwanese schoolchildren showed that the prevalence of AR was associated with levels of SO_2_, CO, and NO_x_, but not with levels of O_3_ and PM_10_ [17]. Another Taiwanese study reported that children’s AR was associated with non-summer warmth and traffic-related air pollutant levels, including CO, NO_x_ and O_3_ [18]. For Asia adults, a cross-sectional population-based study in Singapore found that outdoor air pollution was a significant environmental risk factor of AR [19]. A time-series study identified an association between ambient air pollutant levels and daily outpatient visits for AR among 1506 patients (96% adults) in Beijing [20]. NO_2_ and SO_2_ concentration, but not PM_10_, were associated with increased prevalence of AR among kindergarten children in seven cities in Liaoning Province during 2007–2008 [21]. In addition, a study in Changsha (China) showed that the prevalence of AR in children was significantly positively correlated with age-related accumulative personal exposure of PM_10_, SO_2_, and NO_2_ [22].

Moreover, the short-term effects of air pollutants on human health showed seasonal variations with the change of human activity and meteorological factors [23]. Several studies analyzing seasonal effects of air pollutants were focused on major mortality/morbidity [23,24]. There was only one study conducted on the seasonal effect of air pollutants to the AR patients [25]. Therefore, time series data on air pollution and daily number of outpatient for AR is needed to fill in the blanks.

In this article, we discussed the lag effects of air pollutants (specifically focused on particulate matter) on AR in Northeastern China. We also looked into the seasonal effects of air pollutants on the daily number of outpatients with AR.

## 2. Materials and Methods

### 2.1. Air Pollution and Meteorological Data 

Air quality data for the daily PM_2.5_, PM_10_, O_3_, CO, SO_2,_ and NO_2_ concentrations between 1 January 2013 and 31 December 2015 were provided by the Changchun Municipal Environmental Protection Monitoring Center. The daily data was obtained as average values derived from the hourly data of 10 state-controlled monitoring stations distributed across Changchun, except for O_3_ with a running 8-h mean concentrations (which are averaged with specific hour and the preceding 7 h and the averaging period is stepped forward by one hour for each value). Meteorological factors, such as hourly and daily temperatures, humidity, were obtained from the Weather Underground website (www.wunderground.com).

### 2.2. Daily Number of AR Outpatients

Daily numbers of outpatients for AR symptoms between 2013 and 2015 were obtained from the Departments of Otolaryngology-Head and Neck Surgery, First and Second Hospitals, Jilin University. Both hospitals are Class-Three, Grade A-level tertiary university hospitals located in the central districts in Changchun. They are both comprehensive teaching and researching medical centers. 

The study population includes all outpatients examined by general practitioners and specialties during the study period. AR was defined as symptoms of sneezing or a running, itchy or blocked nose without a cold or flu. The principal diagnosis of allergic rhinitis (ICD-9 code 477) was based on medical history, a physical examination, a standardized questionnaire, and the relevant test (such as, skin prick test).

In order to avoid repeated counting, only one visit per individual patient per day was used as daily visit counts. Subsequent follow-ups within 30 days of the initial visit were excluded. This project was approved by the Ethics Review Board of the two hospitals (2016106). All patients have given written consent to participate in the study. All medical interviewers (general practitioners or nurses) were trained to use uniform examination protocols. Medical records and the respective results were confirmed by the supervisors at each hospital.

### 2.3. Data Analysis

To investigate relationships between AR and ambient air pollution levels, the generalized additive model (GAM) with penalized splines were used to analyze the association of AR with air pollution, adjusting for potential confounders including meteorological factors, time trends, and day of the week. Due to the counted daily outpatients number for AR was small and approximately followed a Poisson distribution [26,27], the core analysis used a GAM with log link and Poisson error that accounted for smooth fluctuations in daily AR patients number. 

Two basic steps needed to be conducted before conducting the model analyses, i.e., development of the best base model without any pollutants and the main model with pollutants. The latter is built by adding the air pollution variables to the final cause-specific best base model, assuming there is a linear relationship between the air pollutant concentration and logarithmic outpatient number.

We initially constructed the basic pattern of outpatients excluding the air pollutants by incorporating smoothed spline functions of time and weather conditions. This makes a flexible modeling tool to include non-monotonic and non-linear links between outpatient visits and time/weather conditions. Next, we considered adding the pollutant variables and further analyzed their effects on AR. To compare the relative quality of the outpatient predictions across these non-nested models, Akaike’s Information Criterion (AIC) was used as a measure of how well the model fitted the data. Smaller AIC values indicate the preferred model. The following formula (log-linear GAM) is fitted to estimate the pollution log-relative rate *β*:

$$\log\left[E\left(Y_{t}\right)\right]=\alpha +\overset{q}{\underset{i=1}{\sum }}\beta_{i}\left(X_{i}\right)+\overset{p}{\underset{j=1}{\sum }}f_{j}\left(Z_{j},df\right)+W_{t}\left(week\right)$$

where $E\left(Y_{t}\right)$ represents the expected outpatient visit number for AR at day t; *β* represents the log-relative risk of outpatient visit associated with an unit increase of air pollutants; $X_{i}$ indicates the concentrations of pollutants at day t; $\sum_{j=1}^{p}f_{j}\left(Z_{j},df\right)$ is the non-parametric spline function of calendar time, humidity, temperature, wind speed and barometric pressure; $W_{t}\left(week\right)$ is the dummy variable for day of the week. More detailed introduction to the GAM has been previously described [27,28].

For the basic models, we also conducted a sensitivity analysis referring to Qian’s method [29] and Welty’s method [30]. We initialized the df as 8 df per year for time, 3 df for temperature, humidity, wind speed, and barometric pressure.

We further examined the effect of air pollutants with different lag (L) structures of single-day lag (distributed lag; from L0 to L7) and multi-day lag (moving average lag; L01 to L07). In this study, a lag of 0 days (L0) means the current-day pollution, and a lag of 1 day corresponds to the previous-day pollution. In multi-day lag models, L03 refers to a 4-day moving average of pollutant concentration of the current and previous 3 days [31]. The meteorological variables used in the lag models were the current day’s data.

For seasonal analysis, seasonality was differentiated on the basis of heating/non-heating periods. In Changchun, the cold (heating) season is from October to April, and the warm (non-heating) season is from May to September. The majority of the heating in Changchun is provided by a central heating of the city from coal burning power plants. In order to avoid the effects of pollen, we classified the warm season as from mid-May to September, and the cold season as from November to mid-April. Air pollution load during the heating season increases significantly compared to non-heating season.

All statistical analyses were conducted using R version 3.1.2 (mgcv package) (all the related data and code are open-source distributed in the Supplementary Materials). Relative risk (RR) was estimated as $e^{\beta \times \Delta C}$, where ΔC is the increased amount of air pollutants. In this study, we used standardized deviation (SD) as the ΔC. We also calculated percent change in the number of consultations for AR patients by (RR-1)*100%. A *p* < 0.05 was considered as statistical significant. All *p* values were 2-sided. 

## 3. Results

### 3.1. General Statistical Analysis

Table 1 summarizes the data for the daily number of AR outpatients and meteorological and air pollution variables in Changchun during 2013–2015. There were 23,344 AR outpatients recorded, with a daily mean admission of 21.7 over this 3-year time-series study period. Age distribution and gender of AR outpatients are also summarized in Table S1 of the Supplementary Materials.

The results showed the daily mean concentrations of PM_10_ and PM_2.5_ were 114.4 μg/m^3^ to 66.5 μg/m^3^ respectively, with both exceeding the yearly concentrations of national level II (70 μg/m^3^ and 35 μg/m^3^). NO_2_ and SO_2_ had a similar trend of monthly mean concentrations, with the concentration of NO_2_ coming in at 43.6 μg/m^3^), near the national level standard (40 μg/m^3^), whereas the concentration of SO_2_ (37.0 μg/m^3^) was also twofold higher than the national level standard (20 μg/m^3^). During the study period, the primary polluting agent in Changchun was the particulate matter emitted from natural and anthropogenic sources, with 27.9% and 20.8% of days PM_2.5_ and PM_10_ pollution, respectively. NO_2_ was second, but with only 3.7% of days above the national standard. During the heating season (November to mid-April) in Figure 1, the concentration of SO_2_ was higher. Concentrations of all air pollutants showed obvious seasonal differences. 

The mean daily temperature and humidity were 6.3 °C and 58.5%, respectively. The mean daily temperature and humidity ranged from −26 °C to 28 °C, and 13% to 90%, reflecting the northern temperate continental monsoon climate of Changchun. Furthermore, extreme lower temperature and atmospheric boundary layer in winter are significant features in northeastern China.

Pearson correlation coefficients of air pollutants and meteorological variables are shown in Table 2. SO_2_, NO_2_, PM_10_, PM_2.5_ and CO had significant positive correlations with each other in both cold and warm seasons (*p* < 0.05), whereas O_3_ had a significant negative correlation with the other five air pollutants in the cold season and only negative correlation with NO_2_ in the warm season. This correlation was consistent with the stationary fossil fuel combustion-related pollutants (SO_2_ and PM) and the traffic-related pollutant NO_2_ [32]. Daily wind speed, daily mean temperature and daily dew point were negatively correlated with all air pollutants except for O_3_ in the cold season. Daily atmospheric press shows a positive relationship with all air pollutants except for O_3_ in both cold and warm seasons. Humidity was negatively correlated with all pollutants except for CO in the warm season, and positively correlated with all pollutants except for PM_10_ and O_3_ in the cold season.

PM_10_ and PM_2.5_ were highly correlated (correlation coefficient r = 0.89) in both the cold and warm season. NO_2_ and SO_2_ were moderately correlated with PM_2.5_ (r = 0.85 and r = 0.64) in cold season, but not in the warm season. CO was highly correlated with PM_2.5_ (r = 0.93 and r = 0.84) in both the cold and warm season due to their acting as byproducts of incomplete combustion, but O_3_ was poorly correlated with PM_2.5_ (−0.23) in cold season and moderately correlated in warm reason, which indicated strong photochemical reactions in summer.

### 3.2. Temporal Patterns of Outpatients and Air Pollutants

**Figure 2:**
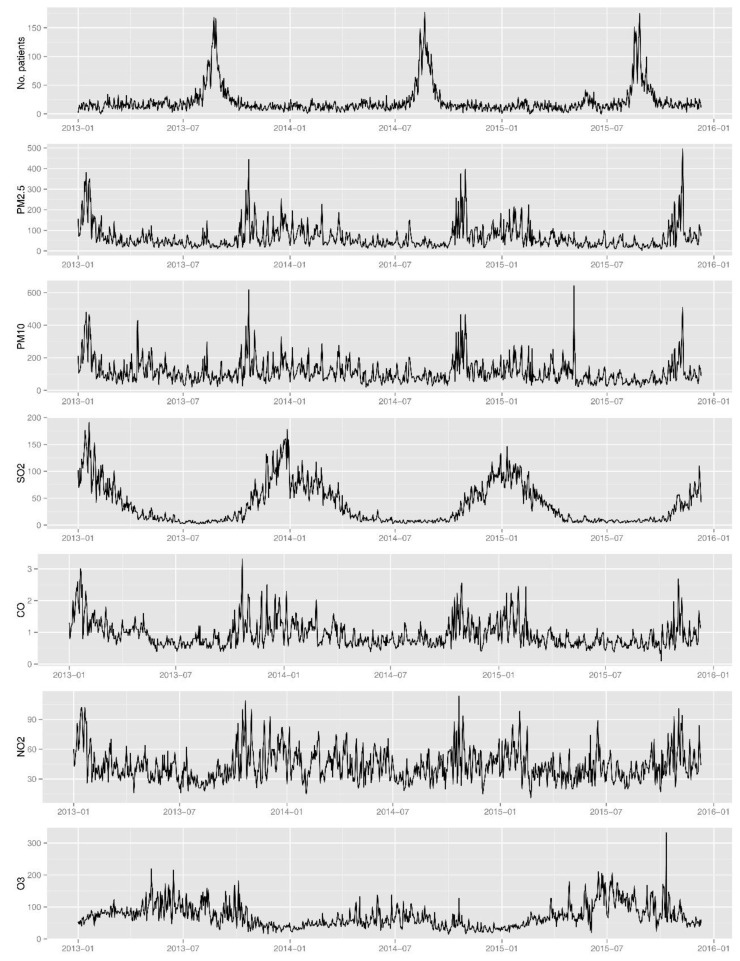
Temporal variations of daily numbers for AR patients and ambient air pollutants (PM_2.5_, PM_10_, SO_2_, CO, NO_2_ and O_3_) in Changchun during 2013–2015.

Figure 2 shows temporal patterns of daily outpatients and daily concentrations of air pollutants in Changchun during 2013–2015. Figure 2 depicts the inter-annual variation of the daily number of AR patients (which ranged from 0 to 177). The mean daily number of AR outpatients was higher in warm than in the cold season. Consistent with the statistical distribution, this number had significant peaks in the warm season, especially around July and September. The number was smaller in other months. This phenomenon could be explained as seasonal allergic rhinitis, which is caused by pollens from weeds and trees [33], such as the pollens emitted from the *Artemisia* and *Ambrosia* in Changchun. 

According to the statistical results, there are obvious seasonal differences for SO_2_, NO_2,_ O_3_ and CO. Although no clear temporal patterns of PM_2.5_ and PM_10_ concentration are evident in Figure 2, there were seasonal differences under further interpretation. PM_10_ had higher daily mean concentrations in spring (May, from dust storms), autumn (October, from straw burning) and winter (November to April, from house heating).

Ranges of PM_2.5_, PM_10_, NO_2_, and O_3_, were wide, with the maximum many times higher than the Class 2 limits of GB3095-2012, according to the national standards of China: GB3095-2012 and HJ633-2012. PM_2.5_ was the major air pollutant in Changchun, and there were a total of 306 days that had heavy fine particulate pollution with daily concentrations higher than 75 µg/m^3^. PM_10_ was the secondary air pollutant, and there were 228 days and 13 days that had heavy PM_10_ and SO_2_ pollutions, with daily concentrations exceeding 150 µg/m^3^, respectively. NO_2_ ranged from 11.2 to 113.5 µg/m^3^, and the annual mean level was 43.6 µg/m^3^. A total of 40 days had heavy NO_2_ pollution with daily concentrations exceeding 80 µg/m^3^. Around 33 days had heavy O_3_ pollution with daily maximum 8-h mean concentrations exceeding 160 µg/m^3^, most of which were during June–October. No polluted days for CO were observed during the study period.

### 3.3. Exposure-Response Associations

**Figure 3:**
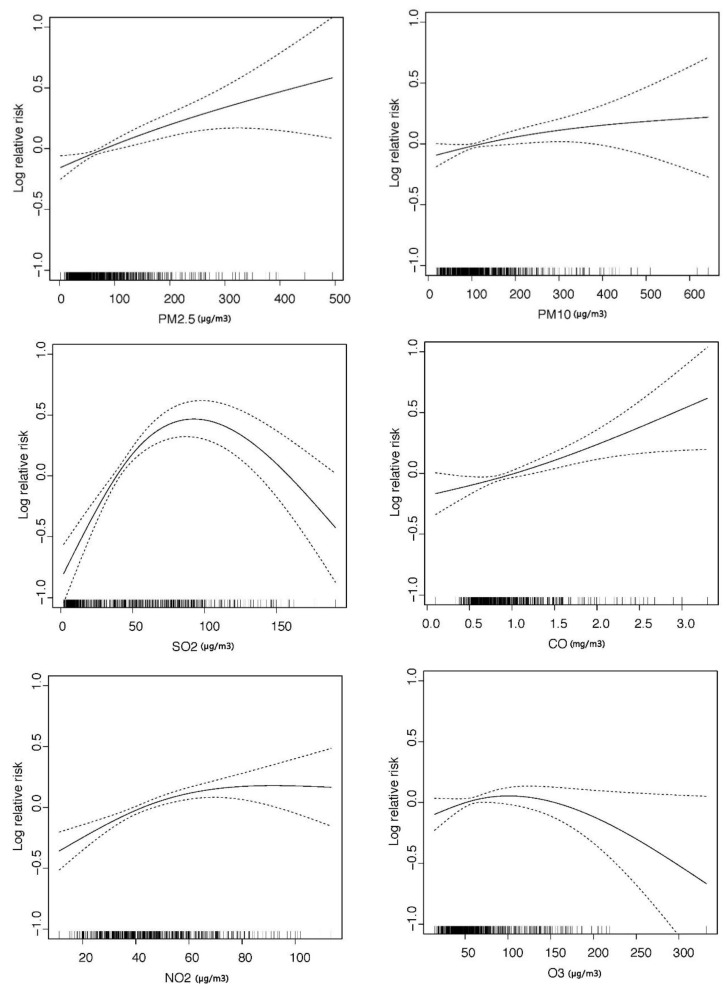
Smoothed plots of exposure-response relations between air pollutants (current day) and outpatient visits for AR in Changchun, China (2012–2015). The solid line presents log relative risk of outpatient visits for AR, while the dashed lines present 95% confidence interval (CI) of the log relative risk.

Figure 3 shows the exposure-response relationships for air pollutants with outpatient visits for AR. In this study, we found generally linear relationships (monotonic trends) for AR hospital visits associated with PM_2.5_, PM_10_ and CO. Moreover, we observed basically monotonic increased relative risk for both SO_2_ and NO_2_ within these ranges of concentrations (Figure 3), indicating that SO_2_ and NO_2_ are significantly associated with increased hospital visits of AR.

**Figure 4:**
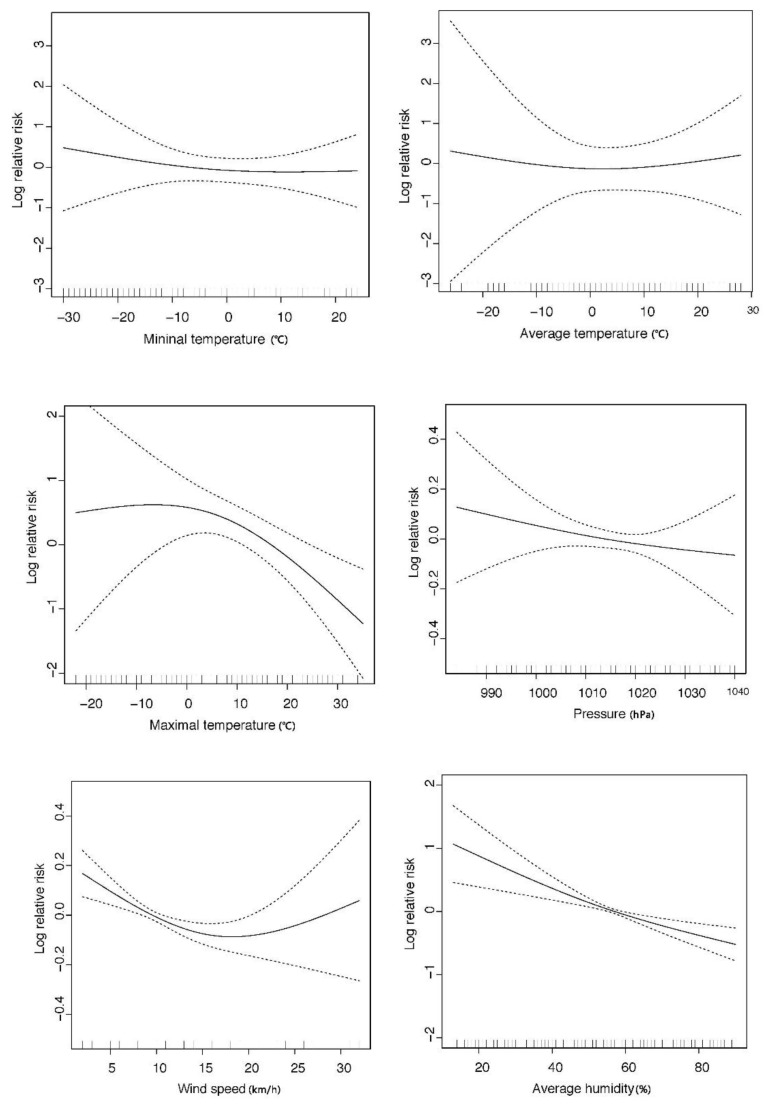
Smoothed plots of exposure-response relations between meteorological factors and outpatient visits for AR in Changchun, China (2012–2015). The solid line presents log relative risk of outpatient visits for AR, while the dashed lines present 95% confidence interval (CI) of the log relative risk.

Figure 4 shows the exposure-response relationships for meteorological factors with hospital visits for AR. The exposure–response relationships associated with humidity and atmospheric pressure were essentially linear with monotonic increases, respectively. The relationships of minimal and maximal temperature were non-linear with higher positive dose-response functions at lower temperatures (<0 °C).

We observed a positive exposure–response relationship between lower wind speeds (<10 km/h) and AR relative risk, which is the result of static wind condition that not suitable for the diffusion of atmospheric pollutants. 

Table 3 illustrates the associations between air pollution and the prevalence of AR in Changchun. It is obvious that high level of PM_2.5_, PM_10_, NO_2_ and SO_2_ were associated with increased hospital visits of AR. Among these pollutants, NO_2_ and PM_2.5_ showed stronger influences on outpatient visits than SO_2_ and PM_10_. Each 1-SD increase of PM_2.5_, PM_10_, SO_2_ and NO_2_ exposure was associated with 10.2% (95% CI, 5.5%–15.1%), 4.9% (95% CI, 0.8%–9.2%), 8.5% (95% CI, −1.8%–19.8%) and 11.1% (95% CI, 5.8%–16.5%) increase of AR prevalence, respectively. No significant associations were observed for CO and O_3_.

### 3.4. Lag Effects of Air Pollutants on AR

The lag effects of air pollutants on the daily number of AR outpatient visits are further analyzed to identify the possible delayed health effects of air pollutants. Given the high correlations between air pollutants, multiple air pollutant exposure effects on AR in one model were not considered in this study.

Table 4 shows the change of RRs in the number of outpatients for AR with each 1-SD increase in pollutants for single-day, 1–7 days prior to the outpatient visit (L0–L6), and moving average measures form day 0 and day 1 to day 7 prior to the visit. For each pollutant, the cumulative measure is mean (lags 0–1; L0–1), mean (lags 0–2; L0–2), mean (lags 0–3; L0–3), mean (lags 0–4; L0–4), mean (lags 0–5; L0–5) and mean (lags 0–6; L0–6), respectively.

The largest RRs of PM_2.5_ for single-day lags were found for the current day (L0), and then decreased to 1.07–1.08 in the following 7 days. The effect magnitude of PM_10_ showed a decreasing trend from L0 to L7. The effect magnitude of SO_2_ for both single-day and multi-day lags showed a decreasing trend from the current day to the third day, but then showed an increasing trend from the third day to the sixth day. The greatest RRs were found in the 1-day cumulative measures (L0–1). RRs of NO_2_ had similar time trends to that of PM_2.5_, and the largest associations were for L0. Then, the RRs decreased to around 1.05 over the next 5 days. Increasing trends for moving average day effects from L0–1 to L0–6 were observed for PM_2.5_, PM_10_, NO_2,_ and CO, whereas there was no association between the current day and lag day of O_3_ with AR. 

## 4. Discussion

This study utilized environmental and epidemiological data to focus on the air quality with temporal variation and its health effects on daily outpatient visits for AR in Changchun during 2013–2015. In contrast to the self-report study conducted in China [34], our study was based on medical records from the two major hospitals in Changchun. We analyzed the daily number of outpatients for AR and attempted to establish time-series relationships between air pollution, metrological factors, and daily outpatient visits for AR. 

Compared to former studies conducted in Beijing [20], which only focused on PM_10_, SO_2_ and NO_2_, all the officially monitored parameters of air pollution and metrology were included in this study (especially adding PM_2.5_, CO and O_3_ as regularly monitored pollutants). Another important difference is that the study period in this paper was extended to 3 years in comparison to former studies, which lasted for only several months to one year [25]. The result shows that daily concentrations of air pollutants had clear seasonal differences. O_3_ levels were higher in the warm season than in the cold season. However, concentrations of the other five pollutants were higher in the cold season than in the warm season. Similar pollution characteristics were reported in northern cities of China, such as Beijing [35] and Shenyang [36]. From June to August, the air quality is relatively better due to the dilution effect of the meteorological conditions. Whereas in the heating period in winter, the air quality is comparatively poor because of the prevailing weather conditions and the lowest PBL height. Although the sources of air pollution sources in the mega cities have gradually changed from conventional coal combustion to a mixture of combustion as well as motor vehicle emissions [37], biomass burning sources in northeastern China still have a tremendous contribution on the heavy air pollution [38]. Moreover, during the periods of March to May and August to October, the concentration of air pollen peaks due to a high pollen release [33]. 

Many studies have reported health effects of PM_2.5_ [39], but O_3_ also has a strong potentially adverse health effects on various respiratory symptoms such as, dyspnea, upper airway irritation, coughing, and chest tightness [40]. However, no significant association between O_3_ and the prevalence of AR was observed in Changchun. Research on harmful effects of O_3_ is rare in China, so further studies are needed. De Marco et al. [41] have reported that outdoor NO_2_ interacted with climate, and increased the risk for AR in high stable temperatures. Hajat et al. [26] found a strong association between 4-day lag SO_2_ and O_3_ and the number of consultations for AR in London. However, PM_10_ and PM_2.5_ are less significant associated with AR in London. Villeneuve et al. [42] reported that there was no statistically significant association between daily levels of air pollution and the number of physician visits for rhinitis among the elderly in Toronto. They only studied SO_2_ and NO_2_ exposure on the same day of the outpatient visits during the winter period. Similar to other time series studies (Table 5), the present work also revealed a statistically significant association between air pollution and AR. RR percentage changes (RRs) in the number of AR outpatients with a 10 µg/m^3^ increase of air pollutants were 2.3%, 6.9%, 1.7% and 0.7% for SO_2_, NO_2_, PM_2.5_ and PM_10_, respectively. In contrast with lag effects of air pollutants in other studies, the highest RRs for SO_2_, NO_2_, PM_10,_ and PM_2.5_ were for the current day and L04 for CO and O_3_. This may be attributed to the differences of exposure risk factors between cities, including environmental factors (geographical location, climatic type and air quality), population (children, adults and elderly) and allergen exposure (time, dose and number). More in-depth studies should be done to clarify the relationship between specific chemical components of particulates (e.g., ions, dust, black carbon, heavy metals, VOCs) and the provenance of AR.

It is of interest and importance to understand the shape of exposure-response association in investigating potential health effects of air pollutants and meteorological factors. In contrast, and like other studies in China [17,20,25], we found nonlinear associations for SO_2_ and NO_2_ that the relative risk increased at the lower SO_2_ and NO_2_ concentrations, but attenuated or even turned negative at higher concentrations (Figure 3). One possible reason is that saturation mechanism, when underlying biochemical and cellular processes become saturated with small doses [43]. Another reason is the small sample size at higher concentrations as can be reflected by the wider confidence intervals. Nonetheless, it should be noted that the ranges of air pollutant concentrations in our study were rather wide, compared with that of the U.S. and Europe. This figure also obviously demonstrated that the current air quality standard of national level II for PM_2.5_ and PM_10_ (daily concentration: 70 μg/m^3^ and 150 μg/m^3^) can effectively reduce the effects to health of AR patients in Changchun. Both the cross-sectional studies and time-series studies (Table 5) have firstly established the significant association between AR and traffic-related NO_x_ emissions. 

A controlled exposure experiment was conducted for seasonal AR patients to exposure in air and NO_x_ (400 ppb, 6 h), and the results illustrated an increase in the number and/or activation state of eosinophils in the nasal mucosa, which in turn could enhance the upper airway response to the inhaled allergen and also exacerbate the disease [44]. Glück and Gebbers [45] also demonstrated that non-specific toxic damage of the nasal epithelium was possibly caused by air pollutants besides grass pollen. The effects of O_3_ on AR were still in debate and were only shown by a few studies [9,11,17]. However, a human exposure experiment suggested that short-term exposure to ozone (250 ppb, 3 h) can increase the bronchial allergen responsiveness in mild allergic asthma or rhinitis patients. Likewise, Peden et al. [46] also reported enhanced nasal inflammatory responses in AR patients after O_3_ exposure. According to Hwang and Jaakkola [47], co-exposure to O_3_ and NO_2_ significantly promoted the release of the cytotoxic protein in the nasal lavage after allergen exposure. No study was concerned with SO_2_ exposure. This reveals that more exposure studies need to be conducted with considerations for different concentration gradients of gaseous pollutants to fix the bottom response concentrations for acute exposure.

More and more studies demonstrated the particulates would exacerbate the AR symptoms, and the pathological studies only focus on the diesel exhaust particles (DEP) and transported desert dust particles [48,49]. However, more detailed chemical components (ions, elements, black carbon, organic carbon, etc.) and their effects on AR need to be further investigated, the results of which could be used to propose reasonable control measures for corresponding emission sources. As mentioned above, there are two major mechanisms may account for the increased incidence of AR in industrialized areas. Firstly, increased concentration of pollutants lead to airway sensitization and responsiveness to allergens. Next, airway responsiveness to allergens may subsequently aggravate symptoms of AR [50].

## 5. Conclusions

The current study provided evidence of the adverse effect of ambient air pollution on AR in northeastern China. We examined the lag effects of air pollutants and the possible differences of warm seasons vs. cold seasons of air pollutants on the daily number of outpatients that were consulting the hospital for treatment for AR over a period of 3 years. The results showed that daily concentrations of air pollutants had obvious seasonal differences. The air pollutants positively correlated with the daily number of AR outpatients, and had lag effects on the daily number of AR outpatients. RR in the number of AR outpatients increased with air pollution level. There are also some limitations to our study. With the continuous rapid urbanization process, more people are becoming exposed to high levels of air pollution. Environmental control and public health strategies should be enforced by the health service policy makers to address this increasingly challenging problem. During haze events, both the health care provider and the public should be given real-time alerts on air quality and other allergy indexes. The affected population should be appropriately advised and treated. Autumn is the most important season when alerts need to be raised regarding the cost of health systems for pollen allergies and air pollution from straw burning in northeastern China.

## Figures and Tables

**Table 1:** Summary of environmental variables and daily number of outpatients for AR in Changchun, 2013–2015.

Variables	Mean	SD	Max.	Min.	Median	IQR
Number of AR Patients	21.7	24.5	177	0	15	12
PM_2.5_ (μg/m^3^)	66.5	59	495	2	47.3	48.4
PM_10_ (μg/m^3^)	114.4	74.4	642.4	19.5	96.5	70.4
SO_2_ (μg/m^3^)	37	36.9	191.3	2.4	19.1	50.6
CO (mg/m^3^)	0.93	0.4	3.3	0.1	0.8	0.46
NO_2_ (μg/m^3^)	43.6	16.1	113.5	11.2	40.9	20.4
O_3_ (μg/m^3^)	71.1	37	332.2	14.2	62.3	44.4
MAXT (°C)	12	14.5	35	−22	15	26
AVET (°C)	6.3	14.3	28	−26	9	27
MINT (°C)	0.7	14.5	24	−30	2	26
Dew (°C)	−1.5	13.9	22	−31	−2	25
Press (hPa)	1015	9.5	1040	984	1014	14
Wind (km/h)	10.9	5.1	32	2	10	8
AVEH (%)	58.5	15.3	90	13	60	22

MAXT: maximum temperature; AVEH: mean temperature; MINT: minimum temperature; Dew: dew point; Press: sea level press; AVEH: mean humidity.

**Table 2:** Pearson’s correlation coefficients among environmental variables in cold season and warm season, Changchun 2013–2015.

	PM_2.5_	PM_10_	SO_2_	CO	NO_2_	O_3_	MAXT	AVET	MINT	DEWP	Press	Wind	AVEH
PM_2.5_	1	0.89 **	0.64 **	0.93 **	0.85 **	−0.23 **	−0.17 *	−0.23	−0.27 **	−0.23 **	0.35 **	−0.14 **	0.09 **
	*1*	0.89 **	0.17 *	0.84 **	0.21 **	0.48 **	0.34 **	*0.36*	0.35 **	0.30 *	0.06 **	0.01 **	−0.00 **
PM_10_		1	0.38 **	0.80 **	0.69 **	−0.018	0.073	0.01 **	−0.06	−0.09 **	0.28 **	−0.03 **	−0.15
		*1*	0.39 **	0.81 **	0.47 **	0.42 **	0.46 **	0.38 **	*0.28*	0.18 **	0.19 **	−0.10 **	−0.26 **
SO_2_			1	0.68 **	0.68 **	−0.58 **	−0.64 **	−0.67 **	−0.65 **	−0.53	0.41 **	−0.28 **	0.46 **
			*1*	0.22 **	0.52 **	0.26 **	*0.05*	−0.08 *	−0.19 **	*−0.42*	0.10 **	0.08 **	−0.64 **
CO				1	0.90 **	−0.30 **	−0.19 **	−0.24 **	−0.27 **	−0.25 **	0.36	−0.12 **	0.07 **
				*1*	0.35 **	0.42 **	0.32 **	0.31 **	0.28 **	0.31 **	*0.21*	−0.17 **	0.10 **
NO_2_					1	−0.36 **	−0.18 **	−0.23 **	−0.27 **	−0.27 **	0.35 **	−0.12	0.01 **
					*1*	*−0.14*	0.22 **	0.09 **	−0.05 **	−0.12 **	0.29 **	*−0.49*	*−0.34*
O_3_						1	0.40 **	0.38 **	0.34	0.31 **	−0.15 **	0.13 **	−0.22
						*1*	0.54 **	0.60 **	0.60 **	0.40 **	−0.31 **	*0.51*	*−0.18*
MAXT							1	0.97 *	0.89	0.83 **	−0.41 *	0.44 *	−0.54 **
							*1*	0.93 **	0.77 **	*0.65*	−0.17 **	−0.02 **	−0.22 **
AVET								1	0.97	0.87 **	−0.48 **	0.50 **	−0.51 **
								*1*	0.94 **	*0.82*	−0.35 **	*0.10*	0.02 **
MINT									1	0.84 **	−0.54 **	0.53 **	−0.46 **
									*1*	0.90 *	−0.48 **	*0.19*	0.23 **
DEWP										1	−0.53 **	0.38 **	−0.06 **
										*1*	−0.37 **	*−0.01*	0.56 **
Press											1	−0.51 **	0.10
											*1*	−0.39 **	−0.17 **
Wind												1	−0.39
												*1*	−0.17 **
AVEH													1
													*1*

* Correlation is significant at the 0.05 level (2-tailed); ** Correlation is significant at the 0.01 level (2-tailed); Italic values are correlation coefficients for the warm season.

**Table 3:** Associations between each 1-SD increase of current day air pollution and allergic rhinitis.

Pollutant	Coefficient β (95% CI)	Relative Risk RR (95% CI)	Percent Change, % (95% CI)
PM_2.5_	0.097 (0.053, 0.140)	1.102 (1.055, 1.151)	10.2 (5.5, 15.1)
PM_10_	0.048 (0.008, 0.088)	1.049 (1.008, 1.092)	4.9 (0.8, 9.2)
SO_2_	0.081 (−0.018, 0.181)	1.085 (0.982, 1.198)	8.5 (−1.8, 19.8)
NO_2_	0.105 (0.057, 0.153)	1.111 (1.058, 1.165)	11.1 (5.8, 16.5)
CO	−0.023 (−0.098, 0.052)	0.977 (0.907, 1.053)	−2.3 (−9.3, 5.3)
O_3_	−0.007 (−0.060, 0.047)	0.993 (0.941, 1.048)	−0.7 (−5.9, 4.8)

**Table 4:** Associations between each 1-SD increase of air pollution with different lag periods and allergic rhinitis prevalence.

Lag	PM_2.5_		PM_10_		SO_2_		NO_2_		CO		O_3_	
	RR	95% CI	RR	95% CI	RR	95% CI	RR	95% CI	RR	95% CI	RR	95% CI
L0	1.102	1.055–1.151	1.049	1.008–1.092	1.085	0.982–1.198	1.111	1.058–1.165	0.977	0.907–1.053	0.993	0.941–1.048
L1	1.071	1.027–1.117	1.029	0.991–1.069	1.036	0.940–1.142	1.048	1.007–1.091	1.008	0.938–1.083	0.975	0.934–1.017
L2	1.085	1.042–1.130	1.048	1.011–1.087	1.004	0.915–1.103	1.043	1.004–1.083	1.026	0.956–1.101	0.983	0.946–1.023
L3	1.081	1.039–1.125	1.049	1.012–1.087	0.984	0.8899–1.078	1.047	1.009–1.086	1.024	0.954–1.099	0.996	0.958–1.035
L4	1.08	1.038–1.123	1.049	1.012–1.088	1.017	0.929–1.113	1.049	1.011–1.087	1.044	0.971–1.121	1.005	0.967–1.044
L5	1.081	1.039–1.126	1.039	1.002–1.078	1.021	0.931–1.120	1.05	1.012–1.089	1.006	0.933–1.085	0.994	0.956–1.033
L6	1.078	1.035–1.121	1.037	1.000–1.076	1.024	0.934–1.124	1.025	0.988–1.064	0.993	0.920–1.073	1.006	0.967–1.045
L0–1	1.113	1.060–1.169	1.053	1.006–1.102	1.086	0.969–1.218	1.109	1.052–1.168	0.992	0.912–1.079	0.971	0.915–1.030
L0–2	1.138	1.080–1.200	1.073	1.021–1.127	1.075	0.950–1.217	1.115	1.055–1.178	1.008	0.920–1.105	0.965	0.908–1.026
L0–3	1.156	1.094–1.222	1.089	1.033–1.149	1.059	0.929–1.207	1.129	1.066–1.197	1.02	0.924–1.125	0.968	0.908–1.032
L0–4	1.171	1.105–1.241	1.104	1.044–1.168	1.065	0.930–1.220	1.143	1.076–1.214	1.037	0.934–1.150	0.974	0.911–1.041
L0–5	1.186	1.117–1.260	1.114	1.049–1.182	1.072	0.931–1.235	1.158	1.088–1.233	1.037	0.929–1.157	0.971	0.906–1.040
L0–6	1.199	1.127–1.277	1.122	1.054–1.194	1.082	0.936–1.252	1.164	1.089–1.243	1.033	0.920–1.160	0.973	0.905–1.046

**Table 5:** RR in the number of outpatients for AR with a 10 µg/m^3^ increase in pollutants for lag effects.

Variables	Toronto [42]		London [26]		Taiwan [17]		Beijing [25]		Changchun	
	Concentration	RR	Concentration	RR	Concentration	RR	Concentration	RR	Concentration	RR
SO_2_ (μg/m^3^)	12.3 (7.3)	1.041	21.2 (7.8)	−0.003	9.2 (5.23)	1.43	44.1 (21.0)	−0.022	37.0 (36.9)	1.023
NO_2_ (μg/m^3^)	47.8 (14.5)	1.018	63.2 (19.7)	1.017	51.9 (15.7)	1.11	52.7 (22.5)	4.804	43.6 (16.1)	1.069
CO (mg/m^3^)	1.3 (0.5)	1	0.9 (0.5)	1.008	0.7 (0.2)	1.05	-	-	1.5 (0.4)	−0.023
O_3_ (μg/m^3^)	59.1 (30.3)	−0.002	34.5 (22.7)	−0.005	45.5 (6.5)	1.05	-	-	71.1 (37.0)	−0.007
PM_2.5_ (μg/m^3^)	7.7 (3.3)	−0.001	12.7 (7.9)	1.037	-	-	-	-	66.5 (59.0)	1.017
PM_10_ (μg/m^3^)	16.4 (4.9)	−0.004	28.5 (13.7)	1.016	55.6 (16.6)	1	116.1 (67.7)	1.367	114.4 (74.4)	1.007
Temperature	8.4 (10.5)		11.9 (5.0)		23.7 (0.8)		12.0 (12.3)		6.3 (14.3)	
Humidity	70.2 (12.1)		70.4 (10.9)		74.0 (3.0)		53.3 (19.3)		58.5 (15.3)	
Period	1995−2000		1992−1994		2000		2009.03–2010.03		2012–2015	
Population	Elderly (≥64 years)		Adults (15−64 years)		Children (6−15 years)		General (all aged)		General (all aged)	

## Supplementary Materials

The following are available online at www.mdpi.com/1660-4601/14/3/226/s1, Table S1: Age distribution and gender of AR outpatients in Changchun; All the related data and code for statistical analyses are open-source distributed in the Supplementary Materials.
