# Supplementary material for: The Association between Ambient Air Pollution and Allergic Rhinitis: Further Epidemiological Evidence from Changchun, Northeastern China
Source: Int J Environ Res Public Health. 2017 Feb 23;14(3):226. doi: 10.3390/ijerph14030226 (PMC5369062; doi:10.3390/ijerph14030226)
Supplement: Supplementary file 1 [file ijerph-14-00226-s001.zip › Supplementary files-done/References of Figure 1.pdf]

No.	Reference
**1**	Chen P C, Lai Y M, Wang J D, et al. Adverse effect of air pollution on respiratory health of primary school children in Taiwan[J]. Environmental Health Perspectives, 1998, 106(6): 331.
**2**	Han Y and Zhang H. Epidemiological investigation of allergic rhinitis in the primary school students in grade three of Shihezi city[J]. Journal of clinical otorhinolaryngology, head, and neck surgery, 2009, 23(23): 1074-1078.
**3**	Huang C, Liu W, Hu Y, et al. Updated prevalences of asthma, allergy, and airway symptoms, and a systematic review of trends over time for childhood asthma in Shanghai, China[J]. PloS one, 2015, 10(4): e0121577.
**4**	Hwang C Y, Chen Y J, Lin M W, et al. Prevalence of atopic dermatitis, allergic rhinitis and asthma in Taiwan: a national study 2000 to 2007[J]. Acta dermato-venereologica, 2010, 90(6): 589-594.
**5**	Jiang M, Wang Q, Li Z, et al. The questionnaire survey of children allergic rhini-tis in Nanjing [J]. Chinese Archives of Otolaryngology-Head and Neck Surgery, 2006, 2: 015.
**6**	Kao C C, Huang J L, Ou L et al. The prevalence, severity and seasonal variations of asthma, rhinitis and eczema in Taiwanese schoolchildren[J]. Pediatric Allergy and Immunology, 2005, 16: 408–415.
**7**	Katelaris C H, Lai C K W, Rhee C S, et al. Nasal allergies in the Asian‐Pacific population: Results from the Allergies in Asia‐Pacific Survey[J]. American journal of rhinology & allergy, 2011, 25(Supplement 1): S3-S15.
**8**	Kong W J, Chen J J, Zheng Z Y, et al. Prevalence of allergic rhinitis in 3–6‐year‐old children in Wuhan of China[J]. Clinical & Experimental Allergy, 2009, 39(6): 869-874.
**9**	Lau Y L, Karlberg J. Prevalence and risk factors of childhood asthma, rhinitis and eczema in Hong Kong[J]. Journal of paediatrics and child health, 1998, 34(1): 47-52.
**10**	Lee Y L, Shaw C K, Su H J, et al. Climate, traffic-related air pollutants and allergic rhinitis prevalence in middle-school children in Taiwan[J]. European Respiratory Journal, 2003, 21(6): 964-970.
**11**	Leung R, Ho P. Asthma, allergy, and atopy in three south-east Asian populations[J]. Thorax, 1994, 49(12): 1205-1210.
**12**	Li A, Sun Y, Liu Z, et al. The influence of home environmental factors and life style on children's respiratory health in Xi'an[J]. Chinese Science Bulletin, 2014, 59(17): 2024-2030.
**13**	Li C W, Chen D H, Zhong J T, et al. Epidemiological characterization and risk factors of allergic rhinitis in the general population in guangzhou city in China[J]. PloS one, 2014, 9(12): e114950.
**14**	Li F, Zhou Y, Li S, et al. Prevalence and risk factors of childhood allergic diseases in eight metropolitan cities in China: a multicenter study[J]. BMC public health, 2011, 11(1): 1.
**15**	Liao P F, Sun H L, Lu K H, et al. Prevalence of childhood allergic diseases in central Taiwan over the past 15 years[J]. Pediatrics & Neonatology, 2009, 50(1): 18-25.
**16**	Liu M M, Wang D, Zhao Y, et al. Effects of outdoor and indoor air pollution on respiratory health of Chinese children from 50 kindergartens[J]. Journal of Epidemiology, 2013, 23(4): 280-287.
**17**	Lu C, Deng Q H, Ou C Y, et al. Effects of ambient air pollution on allergic rhinitis among preschool children in Changsha, China[J]. Chinese Science Bulletin, 2013, 58(34): 4252-4258.
**18**	Ma Y, Zhao J, Han Z R, et al. Very low prevalence of asthma and allergies in schoolchildren from rural Beijing, China[J]. Pediatric pulmonology, 2009, 44(8): 793-799.
**19**	Qian D, Hong S, Yang Y, et al. Survey on epidemiological features of allergic rhinitis in western areas of China [J]. Acta Academiae Medicinae Militaris Tertiae, 2008, 6: 030.
**20**	Song N, Mohammed S, Zhang J, et al. Prevalence, severity and risk factors of asthma, rhinitis and eczema in a large group of Chinese schoolchildren[J]. Journal of Asthma, 2014, 51(3): 232-242.
**21**	Wang T T, Zhao Z H, Yao H, et al. Housing characteristics and indoor environment in relation to children's asthma, allergic diseases and pneumonia in Urumqi, China[J]. Chinese Science Bulletin, 2013, 58(34): 4237-4244.
**22**	Zhang L, Han D, Huang D, et al. Prevalence of self-reported allergic rhinitis in eleven major cities in china[J]. International archives of allergy and immunology, 2009, 149(1): 47-57.
**23**	Zhang Y M, Zhang J, Liu S L, et al. Prevalence and associated risk factors of allergic rhinitis in preschool children in Beijing[J]. The Laryngoscope, 2013, 123(1): 28-35.
**24**	Zhang Y P, Li B Z, Huang C, et al. Ten cities cross-sectional questionnaire survey of children asthma and other allergies in China[J]. Chinese Science Bulletin, 2013, 58(34): 4182-4189.
**25**	Zhao J, Bai J, Shen K, et al. Self-reported prevalence of childhood allergic diseases in three cities of China: a multicenter study[J]. BMC public health, 2010, 10(1): 551.
**26**	Zhao T B, Wang A J, Chen Y Z, et al. Prevalence of childhood asthma, allergic rhinitis and eczema in Urumqi and Beijing[J]. Journal of paediatrics and child health, 2000, 36(2): 128-133.
