# Supplementary material for: The Association between Ambient Air Pollution and Allergic Rhinitis: Further Epidemiological Evidence from Changchun, Northeastern China
Source: Int J Environ Res Public Health. 2017 Feb 23;14(3):226. doi: 10.3390/ijerph14030226 (PMC5369062; doi:10.3390/ijerph14030226)
Supplement: Supplementary file 1 [file ijerph-14-00226-s001.zip › Supplementary files-done/Table S1 Age distribution and gender of AR outpatients in Changchun.pdf]

Table S1. Age distribution and gender of AR outpatients in Changchun.

Age	Male	Female	Total	Proportion of age group%
＜10	1796	947	2743	11.75
10-19	2209	1152	3362	14.40
20-29	2354	2037	4391	18.81
30-39	2230	2322	4552	19.50
40-49	1682	2186	3868	16.57
50-59	1145	1407	2551	10.93
60-69	570	602	1172	5.02
≥70	480	225	705	3.02
Total	12,466	10,878	23,344	100.00
